# The Scope of Drinking Opportunity Creation Is Associated with Stronger Habits and Greater Water Intake in Patients with Kidney Stones

**DOI:** 10.3390/nu18111763

**Published:** 2026-05-30

**Authors:** Ian Kim, Necole M. Streeper, James Marks, David E. Conroy

**Affiliations:** 1 Department of Psychology, Stanford University, Stanford, CA 94305, USA; 2 Department of Urology, Medical College of Wisconsin, Milwaukee, WI 53226, USA; nstreeper@mcw.edu; 3 Department of Urology, Penn State Health Milton S. Hershey Medical Center, Hershey, PA 17033, USA; jamestmarks1@gmail.com; 4 School of Kinesiology, University of Michigan, Ann Arbor, MI 48109, USA; deconroy@umich.edu

**Keywords:** urolithiasis, drinking, water intake, preparatory behavior, automaticity, behavior mechanisms

## Abstract

**Background/Objectives:** Kidney stone patients struggle to attain the recommended fluid intake. Prior work has focused on the strength of habits (i.e., context–behavior associations) for fluid intake, but given the variability in the contexts of daily life, the scope of efforts to create opportunities to drink across contexts may also be important. **Methods**: A cross-sectional study was conducted among adults with a history of kidney stones (*N* = 265). Participants identified situations in which they made an effort to have a glass of water nearby (opportunity creation), rated the experienced automaticity of water intake (i.e., habit strength, measured via the Self-Report Behavioral Automaticity Index), and reported past-week fluid intake volumes. Latent class analysis was used to identify distinct subgroups based on the contexts in which individuals created opportunities to drink, and multivariable linear regression was used to examine the associations between habit strength, class membership, and daily fluid intake. **Results**: Three latent classes were identified based on the scope of opportunity creation across contexts: widespread (27.9% of the sample; water intake: 41.2 ± 17.1 fl oz), selective (43.4%; water intake: 32.6 ± 16.33 fl oz), and limited (28.7%; water intake: 19.01 ± 16.08 fl oz). The widespread class reported stronger habits (22.45 ± 6.43) and higher water intake than the selective (19.97 ± 6.20) or limited classes (14.38 ± 6.81) (all Ps < 0.001). Stronger habits significantly predicted higher daily water intake (b = 0.90, SE = 0.16, *p* < 0.001). No significant association was found between habit strength and total fluid intake volume (b = 1.06, SE = 0.74, *p* = 0.17). **Conclusions**: Habit strength positively predicted water intake for all classes. To increase fluid intake, clinical interventions should help patients develop drinking habits tied to specific daily contexts.

## 1. Introduction

Kidney stones affect a large proportion of the population, with a lifetime prevalence of 11% in men and 7% in women [1]. The stone recurrence rate is high, with repeat episodes in 40% of stone formers within 5 years from the initial episode and 80% within 10 years [2,3]. In addition to pain and suffering, the annual medical care costs for kidney stones in the United States exceeded $2 billion, with additional unmeasured costs due to lost productivity [4,5]. Increasing urine volume and fluid intake is an established approach for decreasing the risk of recurrent stone formation [6,7]. Unfortunately, less than 50% of stone formers consume enough fluids to meet clinical guidelines for reducing recurrence risk [8,9,10,11]. Behavioral interventions can target people’s capabilities, opportunities, or motivation to increase fluid intake [12]. However, little is known about how the efforts an individual makes to create opportunities for fluid intake impact their fluid intake motivation (e.g., habit strength) or behavior. This study investigated the role of opportunity creation in supporting both water intake habits and reported water intake.

Opportunities to drink can be created by making efforts to have water or other fluids nearby. Individuals who have water nearby more often are expected to consume greater amounts, as the presence of accessible water provides greater opportunities for intake. People are mobile, traveling through different spatial contexts throughout the day, so creating more opportunities (i.e., across different contexts) through preparatory behaviors may be even more important for promoting subsequent fluid intake. A recent qualitative study revealed that people who drink water in a wide variety of situations had greater and more consistent water intake than those who situate their drinking in a narrow range of situations [13,14]. Following qualitative evidence, we hypothesized that people who create more opportunities to drink water will consume more water.

The consistency with which an individual creates opportunities to drink in different daily contexts may also support habit formation. Initially, behaviors require opportunities along with conscious effort, planning, and intention, but with continued repetition and reinforcement in specific contexts, those execution behaviors can become more automatic within those specific contexts [15,16,17,18]. Habitual behaviors are driven by impulses triggered when individuals encounter cues that they have learned to associate with specific behavioral responses [19]. As habits form from having previously and repeatedly enacted those behaviors in the presence of those cues, habitual behaviors can be effortlessly initiated by cues without conscious thought or intention. Individuals with stronger habits are more likely to perform associated behaviors when confronted with relevant cues [15,20,21]. Of course, in the context of fluid intake, that proposition assumes that the person has an immediate opportunity to drink. We posit that people who deliberately create these opportunities—such as keeping water nearby across multiple settings—will not only consume more fluids but will also develop stronger intake habits.

One of the challenges in testing these hypotheses is that people have widely varying daily routines, so it may not be possible to identify specific contexts that support habit formation or water intake. Thus, we adopted a person-centered approach to identify patterns of opportunity creation across common daily contexts. Identifying latent classes of opportunity creation patterns accommodates the idiosyncrasies of people’s lived experiences and may reveal key patterns for future interventions to target. This person-centered approach has not been applied previously to examine how the scope of opportunity creation across contexts is associated with habit strength or fluid intake.

This study investigated patterns by which people create opportunities to drink water and their role in supporting habits for water intake and actual water intake in a sample of patients with kidney stones. We applied latent class analysis to identify common and potentially idiographic, contextual patterns of opportunity creation. Although we did not hypothesize the existence of specific profiles, we expected that extensive opportunity creation across multiple contexts would adaptively promote both habit strength and actual water intake. Thus, we hypothesized that patients in classes that reported creating more opportunities to drink water across contexts would have stronger water intake habits and consume more water than people who create fewer opportunities. We also sought to further validate habit strength as a predictor of water and total fluid intake in regression analyses, hypothesizing a positive association between habit strength and water intake for participants regardless of class membership.

## 2. Materials and Methods

### 2.1. Study Design and Ethical Approval

This study used a cross-sectional survey design to examine opportunity creation, habit strength, and fluid intake among kidney stone patients. The study was conducted in accordance with the Declaration of Helsinki and approved by the Institutional Review Board of Penn State University (STUDY00018175 on 22 July 2021). All participants provided informed consent prior to completing the survey.

### 2.2. Participants and Recruitment Procedure

Patients (*N* = 3305) with a history of kidney stones who provided consent to be contacted about research opportunities were identified from electronic health records and sent an email invitation to complete an online survey. The inclusion criterion was adults (age 18+ years) currently residing in the United States with a prior diagnosis of symptomatic kidney stones. Participants were excluded if they were not willing to complete the 30-min survey. Between August and September 2021, 265 patients (8.02% response rate) completed the survey.

### 2.3. Measures

Demographic characteristics that were assessed included age, sex at birth, race, ethnicity, highest educational attainment, and employment status. Standardized questions from the PhenX toolkit were used [22]. Kidney stone history was assessed with three questions: “At what age were you first diagnosed with kidney stones?”, “How many times have you passed a kidney stone to date (to your best estimate)?”, and “How many surgeries have you had to treat your kidney stones (to your best estimate)?”

The scope of efforts to create opportunities to drink water was assessed with a checklist of 19 common contexts in which people might drink (e.g., eating breakfast, sitting to work, using a smartphone, exercising, doing chores). Participants were asked to check each situation where they typically made an effort to have a glass of water nearby. The checklist was developed collaboratively by urologists and behavioral scientists with expertise in kidney stone prevention and health behavior, with items designed to capture common daily contexts for fluid intake based on clinical experience and behavioral theory. The items were iteratively reviewed within the study team to ensure relevance and coverage of typical drinking contexts.

The Self-Report Behavioral Automaticity Index (SRBAI) [23], a 4-item subscale of the Self-Report Habit Index [24], was used to assess the strength of participants’ habits for water intake. Items had the stem, “Drinking tap or bottled water ….” Participants rated each item on a scale ranging from 1 (*strongly disagree*) to 7 (*strongly agree*), and responses were summed into a single scale score. Higher SRBAI scores indicated greater automaticity. Responses had high internal consistency (Cronbach’s α = 0.95).

The Beverage Intake Questionnaire (BEV-Q-15) was used to assess past-week water and fluid intake. Participants rated the frequency (how often) and volume (how much each time) of daily consumption for 15 beverage types using standardized categorical response options. Average daily intake (fl oz/day) was calculated for each beverage, as well as for flavored beverages (e.g., juices, milk, sugar-sweetened and diet drinks, tea, and sport drinks), for water, and for total fluid intake.

### 2.4. Statistical Analysis

Descriptive statistics of demographic variables were calculated using the R package *gtsummary* v2.2.0 [25]. Latent class analysis was used to identify distinct classes defined by the scope of contexts in which a person created opportunities for drinking by typically having water nearby, the relative size of each class (proportion of patients within each class), and the distribution characteristics within each class (probability of each of the nineteen items based on class membership) [26,27]. Prior to conducting the latent class analysis, an exploratory factor analysis based on a tetrachoric correlation matrix supported a unidimensional structure, with all 19 items loading significantly onto a single factor (loadings: 0.38–0.75), accounting for 39.7% of the variance. To select the appropriate number of classes and maximize model fit, a two-class model was first fit to the data and compared with models that specified progressively more latent classes (up to five classes). To ensure that the maximum likelihood solution was correctly identified within these models, 100 iterations of each model (i.e., from two to five classes) were run using randomly generated seed values. The resulting Likelihood Ratio Statistic (G^2^) values were compared across the 100 iterations; the dominant solution (i.e., that which most frequently yielded the same G^2^ values using the randomly generated seeds) was identified as the maximum likelihood solution. Lower G^2^ scores indicate a better fit. In selecting the final model solution, we specifically examined the Akaike Information Criterion (AIC), the Bayesian Information Criterion (BIC), and entropy values across models. Lower AIC and BIC indicate a better fit, and the BIC is believed to perform better than the AIC [28,29,30]. Entropy is a measure from 0 to 1 of how well individuals are assigned to latent classes (class differentiation), with values above 0.80 being acceptable. All models were run and compared using the R package *poLCA* v1.6.0.2 [31].

Mean differences in the strength of water intake habits and daily water and fluid intake volumes were compared across identified subgroups using ANCOVA with Bonferroni-adjusted post hoc pairwise comparisons, while accounting for the effects of demographic characteristics (i.e., age, sex, and education). Associations between habit strength for water intake and daily water/total fluid intake volumes were tested in multivariable linear regressions using the R package *stats* v4.5.0, with potential moderator effects of the latent classes. All models were adjusted for age, sex, employment status, and education.

To determine the required sample size, an a priori power analysis was conducted. For latent class analysis, assuming a medium-to-large effect size across the 19 indicators and a desired power of 0.80, the required minimum sample size was estimated to be 220 [32]. For multiple linear regression models, with a significance level of α = 0.05, desired power (1 − β) = 0.80, and nine predictors (including demographic variables, latent classes, and habit strength), a minimum sample size of *N* = 207 was required to detect a small-to-medium effect size (f^2^ = 0.04). The final sample size of 265 participants exceeded these requirements [33].

## 3. Results

The sample (*N* = 265) was predominantly female (57%), White (97%), and non-Hispanic/Latino (98%). The mean (±SD) age was 55.5 (±15.1) years. Patients reported an average of 5.2 (±5.1) previous kidney stones and 2.8 (±2.6) previous surgeries to treat kidney stones. Mean (±SD) daily water intake and daily total fluid intake were 31.4 (±18.4) fl oz and 57.7 (±28.3) fl oz, respectively, and did not significantly differ between recurrent and non-recurrent stone formers. Table 1 summarizes the demographic and clinical characteristics of the sample.

### 3.1. Latent Classes of Patients Based on Patterns of Creating Opportunities for Drinking Water

**Figure 1:**
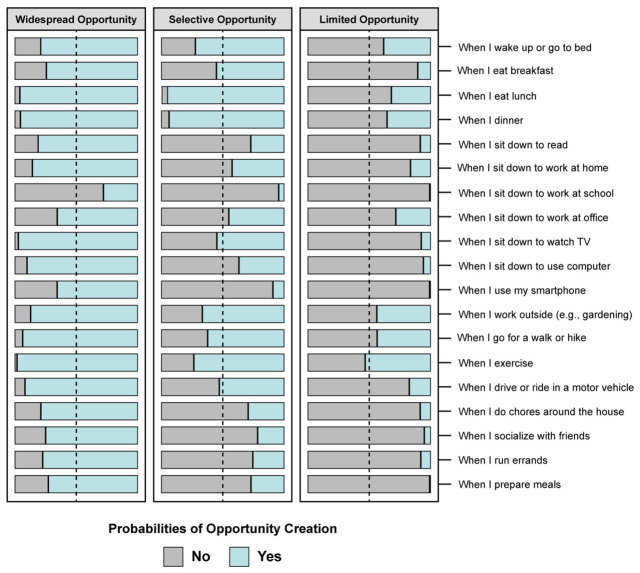
Probabilities of opportunity creation in each context by the identified three latent classes. Dashed lines indicate 50%.

The fit statistics for evaluated latent class solutions are presented in Table 2. Based on these criteria, the three-cluster model was selected as the optimal solution, providing the best balance of model parsimony and classification accuracy (i.e., the lowest BIC and the highest entropy). Table 3 and Figure 1 provide a detailed breakdown of the endorsement probabilities that define these three latent classes.

The first group, Class 1: Widespread Opportunity Creation (27.9%, *n* = 74), was characterized by a “universal” planning strategy. These participants were highly likely to effortfully prepare water to drink in nearly all contexts of daily life. In contrast, Class 2: Selective Opportunity Creation (43.4%, *n* = 115) represented a more context-dependent pattern. These participants were likely to create opportunities to drink water while eating or engaging in structured physical activities (e.g., gardening, exercise, walking, or hiking); however, they were unlikely to prepare opportunities to drink while sitting or engaging in activities of daily living (e.g., doing chores around the house, running errands, or preparing meals). Finally, Class 3: Limited Opportunity Creation (28.7%, *n* = 76) displayed a minimal planning profile. With the sole exception of structured exercise, these participants rarely endorsed any opportunity creation, showing the lowest probabilities across all 19 items compared to the other two classes.

### 3.2. Class Differences in Water Intake Habits and Water and Fluid Intake Volume

**Figure 2:**
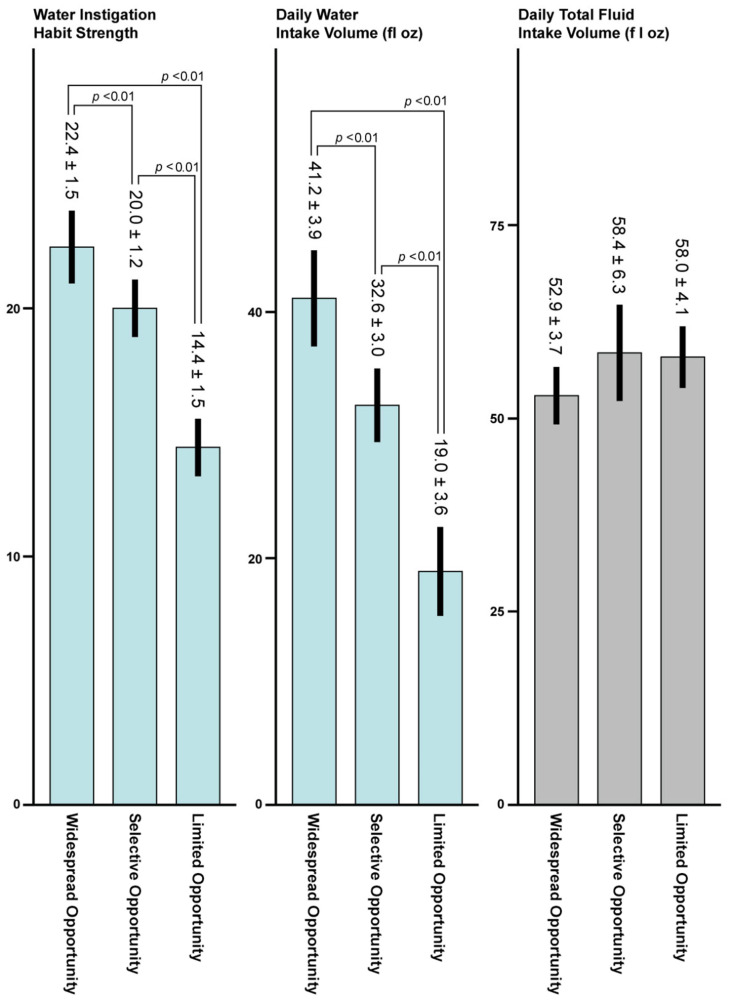
Mean differences with 95% confidence intervals in instigation habit strength for water intake, daily water intake volume, and total fluid intake volume between the three latent classes identified based on the scope of opportunity creation. *p*-values were obtained from pairwise comparisons.

The three latent classes did not differ in age, race, ethnicity, employment status, education level, or medical history. The three latent classes differed significantly in terms of habit strength for water intake (F[2, 262] = 29.27, *p* < 0.001) and in daily water intake volume (F[2, 262] = 31.28, *p* < 0.001). Participants in the Widespread Opportunity Creation class reported significantly stronger habits for water intake (22.45 ± 6.43) and greater daily water intake volume (41.18 ± 17.13 fl oz) than those in the Selective (SRBAI 19.97 ± 6.20 [Cohen’s *d* = 0.39]; water volume 32.56 ± 16.33 [Cohen’s *d* = 0.52]) or Limited Opportunity Creation (SRBAI 14.39 ± 6.81 [Cohen’s *d* = 1.28]; water volume 19.01 ± 16.08 [Cohen’s *d* = 1.33]) classes (all *P*s < 0.001 between pairs). Total fluid intake did not vary significantly across the three classes. The mean differences, 95% confidence intervals, and pairwise comparisons for these outcomes are illustrated in Figure 2.

### 3.3. Habit Strength as a Predictor of Water and Fluid Intake

After adjustment for age, sex, employment status, and education level, water intake volumes were positively associated with habit strength for water intake (b = 0.90, *p* < 0.001), but total fluid intake volumes were not positively associated with habit strength for water intake. Class membership did not moderate the associations between habit strength and either water or total fluid intake volumes. The detailed regression coefficients and model statistics for these analyses are presented in Table 4.

## 4. Discussion

This study investigated how the scope of contexts in which patients with a history of kidney stones create opportunities to drink water is associated with habit strength for water intake, as well as actual water and fluid consumption. As expected, patients varied in the scope of their opportunity creation to drink water in their daily lives, and this scope was associated with stronger habits and greater water intake, representing a more than twofold difference in volume between the highest- and lowest-opportunity-creation groups. These results highlight the dual importance of opportunities and motivation, making three specific contributions to the literature.

First, this study provides the strongest evidence to date for habitual regulation of water intake. Habits have been validated as influences on a variety of health behaviors, including dietary behavior [15]. Although prior work has established that interventions can increase habit strength for water intake [34,35], this study extended the literature by providing the first evidence linking habits for water intake with reported water intake specifically. It also revealed the specificity of habits for water intake, as they were weaker (and not statistically significant) predictors of total fluid intake compared to water intake specifically. Habits for water intake do not appear to generalize well to other behaviors, even similar behaviors. Relations between habit strength and water intake were also consistent across classes. Thus, interventions to support patients in increasing fluid intake should focus on specific drink types or adopt multi-pronged strategies for forming habits across multiple drink types. For individuals with relatively uniform beverage patterns, strategies could focus on strengthening those beverage-specific routines (e.g., for individuals who predominantly consume water, preparing or refilling water bottles in advance during periods of limited access). For individuals with more varied beverage consumption, strategies could leverage individual preferences to suggest appropriate alternatives (e.g., linking water intake to arriving at the workstation, unsweetened iced tea with lunch, and decaffeinated herbal tea to the evening wind-down). Digital interventions are particularly well-suited to support these strategies by prompting preparatory and consumption behaviors in an adaptive, context-aware manner based on time, location, and/or routine patterns.

Second, this study features a novel analysis of the opportunities that patients create, identifying three latent classes. By utilizing latent class analysis, we effectively revealed that preparatory behavior is not a uniform trait but is organized into distinct contextual patterns—a nuance obscured by traditional variable-centered averages. Specifically, the identified classes varied primarily in the scope of efforts to prepare opportunities to drink. This result highlights the importance of preparatory actions preceding a target behavior and provides valuable insights into how action planning can be effectively used to achieve recommended levels of fluid intake among patients with kidney stones. Action planning is a widely employed intervention approach across various domains, commonly involving the identification of specific contexts for enacting desired behaviors [36,37]. Surprisingly, in the context of fluid intake behavior, although action planning has been utilized in mobile applications to promote fluid consumption [38], there is a notable absence of intervention studies that have targeted preparatory behaviors [39]. Interventions may benefit from an expanded scope of planning to increase opportunities for developing fluid intake habits and promoting fluid intake volume.

Lastly, the findings of our study provide support for previous research on situated water intake, suggesting that water intake behaviors are influenced, at least in part, by habits developed within specific contexts [13,14]. Our study extends the literature by quantifying the associations between the scope of opportunity creation across contexts and the strength of habits and volume of water intake, highlighting the role of opportunity creation in establishing supportive contexts to promote water intake. Recently, distinct phases of habit have been proposed: preparatory and instigation/execution habits, with each phase requiring distinct and repeated behavior practice [40,41]. However, empirical evidence distinguishing these phases remains limited. Our finding that the link between opportunity creation (a preparatory act) and water intake habit was not moderated by class membership provides empirical support for this distinction. Although conceptually distinct, repeated engagement in preparation may fuel both phases. Preparatory behaviors may themselves become increasingly automatic over time, while simultaneously increasing the availability and salience of contextual cues that trigger drinking behavior and reduce the cognitive load required to initiate intake. Preparatory routines thus function not only as practical supports, but also as behavioral mechanisms through which both preparatory and instigation habits are reinforced. Clinicians may leverage this interplay to design more comprehensive interventions that address both phases. For instance, rather than simply advising patients to increase fluid volume, clinicians may encourage them to develop consistent routines that create opportunities to drink in specific contexts, such as keeping a glass of water on their desk, to promote preparatory habits, while implementing personalized goals for daily fluid intake volume to support instigation habits.

Several key limitations should be noted. The sample was recruited from a single health system in south-central Pennsylvania (USA) and was predominantly non-Hispanic and White; therefore, findings may not generalize to more diverse samples or samples in other locations. The cross-sectional design precludes causal inferences; future research should test causal hypotheses using longitudinal and experimental designs. All data relied on self-reports, which are susceptible to recall errors and bias; incorporating objective measures (e.g., using sensors to track fluid intake and 24-h urine volume) would strengthen future work. While the opportunity creation checklist was developed with expert clinical input, it lacks formal psychometric validation; future studies may establish comprehensive construct validity and test–retest reliability. Finally, dietary factors, beverage composition, medical conditions, and medication use were not assessed in this study, all of which could influence kidney stone formation and represent potential sources of residual confounding.

In sum, the scope of opportunity creation to drink water across daily contexts supports both stronger habits and higher water intake. Patients who were most consistent in having water available during sedentary activities reported the most automatic and greatest volume of water intake. Efforts to increase fluid intake in patients with kidney stones should focus on developing effective situated drinking habits.

## Figures and Tables

**Table 1:** Demographic, motivational, and behavioral characteristics of the sample.

Characteristics (*N* = 265)	*N* (%) or Mean (SD)
Sex assigned at birth	
Female	150 (57%)
Male	114 (43%)
Prefer not to answer	1 (0.4%)
Age (years)	55.5 (15.1)
Race	
Asian	1 (0.4%)
Black or African American	3 (1.1%)
White	256 (97%)
Two or more races	3 (1.1%)
Other	1 (0.4%)
Hispanic	
Yes	4 (1.5%)
No	261 (98%)
Employment	
Employed, full-time	130 (49%)
Employed, part-time	23 (8.7%)
Retired	92 (35%)
Student	1 (0.4%)
Unemployed	18 (6.0%)
Education	
High school or less	40 (15.5%)
Some college	84 (31.7%)
Bachelor’s degree	70 (26%)
Master’s degree	51 (19%)
Doctoral degree	20 (7.5%)
Do you currently have kidney stones?	
Yes	30 (11%)
No	1 (0.4%)
Unsure	234 (88%)
Age at first diagnosis with kidney stones (years)	18.8 (15.4%)
Number of passing kidney stones to date	5.2 (5.1)
Number of past surgeries to treat kidney stones?	2.8 (2.6)
Habit strength for automatic water intake	19.1 (7.1)
Daily water intake (fl oz)	31.4 (18.4)
Daily total fluid intake (fl oz)	57.7 (28.3)

**Table 2:** Summary of latent class cluster models.

	G^2^	AIC	BIC	Entropy
**2-Cluster**	−2758.24	5594.49	5734.10	0.85
**3-Cluster**	−2664.54	5447.09	5658.29	0.84
**4-Cluster**	−2617.90	5393.80	5676.60	0.84
**5-Cluster**	−2582.32	5362.64	5717.03	0.87

**G^2^:** Log-Likelihood; **AIC**: Akaike information criterion; **BIC**: Bayesian information criterion.

**Table 3:** Probabilities of opportunity creation across contexts by the identified three latent classes.

Creating Opportunities for Water Consumption	Total % Endorsed	Class 1 (*n* = 74)	Class 2 (*n* = 115)	Class 3 (*n* = 76)	Wald	*p*-Value
01. When I wake up or go to bed	63.77	78.38	70.43	39.47	28.47	<0.001
02. When I eat breakfast	47.17	70.27	55.65	11.84	57.23	<0.001
03. When I eat lunch	76.23	93.24	94.78	31.58	117.28	<0.001
04. When I eat dinner	76.60	93.24	93.91	34.21	106.87	<0.001
05. When I sit down to read	36.60	79.73	26.96	9.21	88.50	<0.001
06. When I sit down to work at home	46.79	85.14	42.61	15.79	73.85	<0.001
07. When I sit down to work at school	10.19	27.03	5.22	1.32	32.57	<0.001
08. When I sit down to work at my office	46.04	66.22	45.22	27.63	22.52	<0.001
09. When I sit down to watch TV	52.83	95.95	53.91	9.21	113.28	<0.001
10. When I sit down to use the computer	42.64	89.19	36.52	6.58	107.73	<0.001
11. When I use my smartphone	22.26	63.51	9.57	1.32	102.74	<0.001
12. When I work outside (e.g., gardening)	65.66	86.49	66.96	43.42	30.99	<0.001
13. When I go for a walk or hike	65.28	91.89	63.48	42.11	40.30	<0.001
14. When I exercise	73.96	97.30	73.91	51.32	41.16	<0.001
15. When I drive or ride in a motor vehicle	53.21	90.54	52.17	18.42	78.41	<0.001
16. When I do chores around the house	36.98	78.38	27.83	10.53	81.38	<0.001
17. When I socialize with friends	31.70	72.97	22.61	5.26	87.15	<0.001
18. When I run errands	34.72	75.68	25.22	9.21	81.17	<0.001
19. When I prepare meals	32.08	71.62	26.96	1.32	87.51	<0.001

**Table 4:** Regression coefficients from multivariable linear regression analyses for effects of automaticity of water intake and class memberships on daily water and fluid intake volumes.

	Daily Water Intake Volume (fl oz)		Daily Fluid Intake Volume (fl oz)	
Predictor	b	*p*	*b* [95% CI]	*p*
Intercept	18.75 [4.70, 32.80]	0.009	36.22 [−46.19, 118.63]	0.373
Automaticity for Water Intake	0.90 [0.59, 1.21]	<0.001	1.06 [−0.48, 2.59]	0.168
Latent Class Membership				
Widespread Opportunity Creation (Ref.)	-	-	-	-
Selective Opportunity Creation	−7.50 [−12.44, −2.56]	0.003	−0.27 [−38.85, 38.31]	0.989
Limited Opportunity Creation	−16.00 [−21.98, −10.02]	<0.001	8.79 [−35.08, 52.66]	0.683
Age (years)	0.02 [−0.15, 0.19]	0.813	−0.03 [−0.86, 0.80]	0.947
Sex				
Female (Ref.)	-	-	-	-
Male	2.99 [−1.51, 7.39]	0.182	6.59 [−18.03, 31.21]	0.586
Employment Status				
Unemployed (Ref.)	-	-	-	-
Employed	3.84 [−0.86, 8.54]	0.109	−14.49 [−40.16, 11.18]	0.256
Education				
High school of less (Ref.)	-	-	-	-
Some College	−4.71 [−11.45, 2.03]	0.170	−7.09 [−44.53, 30.34]	0.699
Bachelor’s degree	−4.61 [−11.42, 2.21]	0.184	11.49 [−25.26, 48.07]	0.527
Master’s degree or higher	−6.13 [−13.09, 0.83]	0.084	−4.16 [−43.24, 34.92]	0.828
Model fit	R^2^ = 0.34	<0.001	R^2^ = 0.251	0.544

## Data Availability

The data that support the findings of this study are available on request from the corresponding author. The data are not publicly available due to privacy or ethical restrictions.
